# Supplementary material for: Hybrid Digital-Droplet Microfluidic Chip for Applications in Droplet Digital Nucleic Acid Amplification: Design, Fabrication and Characterization
Source: Sensors (Basel). 2023 May 20;23(10):4927. doi: 10.3390/s23104927 (PMC10221416; doi:10.3390/s23104927)
Supplement: Supplementary file 1 [file sensors-23-04927-s001.zip › sensors-2370111-supplementary.pdf]

sensors

Hybrid Digital-Droplet Microfluidic Chip for Applications in Droplet Digital Nucleic Acid Amplification: Design, Fabrication and Characterization – Supplementary Material

Beatriz J. Coelho[1,2,†], Joana P. Neto[1,†], Bárbara Sieira[1], André T. Moura[1], Elvira Fortunato[1], Rodrigo Martins[1], Pedro V. Baptista[2,*], Rui Igreja[1,*] and Hugo Águas[1,*]

[1] CENIMAT|i3N, Department of Materials Science, NOVA School of Science and Technology, Campus de Caparica, NOVA University of Lisbon and CEMOP/UNINOVA, 2829-516 Caparica, Portugal
[2] UCIBIO, I4HB, Department of Life Sciences, NOVA School of Science and Technology, Campus de Caparica, NOVA University of Lisbon, 2829-516 Caparica, Portugal
* Correspondence: pmvb@fct.unl.pt (P.V.B.); rni@fct.ul.pt (R.I.); hma@fct.unl.pt (H.Á.)
† These authors contributed equally to this work.

Supplementary Information S1

Close-up on the DrMF section of the hybrid devices and DrMF standalone devices

Figure S1.1 illustrates the structure of the DrMF portion of the developed devices, evidencing all the relevant dimensions. Briefly, the DrMF section of the hybrid devices includes two access points for the two phases required to create nano-liter droplets at the flow-focusing region (region highlighted in blue). The oil phase is inserted in the circular inlet (region highlighted in red) and flows laterally both right- and leftwards, down to the flow-focusing area. The aqueous phase enters via the DMF section of the hybrid device, which is not shown but is located below the droplet generation/flow-focusing area. The aqueous samples experience a pulling force exerted by a syringe pump connected to the tear-shaped access point, located below the circular inlet highlighted in red.

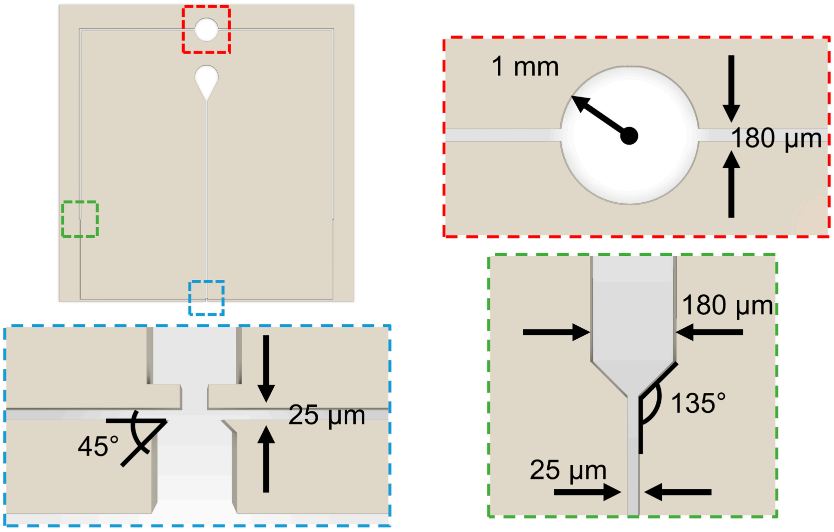

Figure S1. 1: Representation of the DrMF section of the hybrid devices, evidencing relevant features and dimensions. The flow-focusing region (highlighted in blue, bottom left) receives the aqueous phase from the DMF section located below the represented device, and the oil phase from the left and right channels. The access point for the oil phase is represented on the top right (highlighted in red), and the channel width reduction zone is represented on the bottom right (highlighted in green).

Figure S1.2 further illustrates a top view of standalone DrMF devices, evidencing the differences compared to the DrMF section of hybrid devices. Basically, there is an additional piece in the PDMS, which contains a reservoir for LAMP buffer supply.

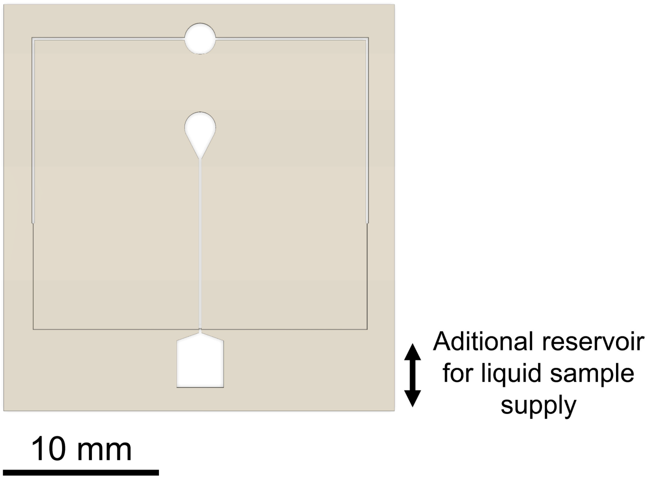

Figure S1. 2: Schematic illustration of the standalone DrMF devices (top view), evidencing the additional reservoir present in the PDMS.

Supplementary Information S2

Channel width variation after deposition of Parylene C and PTFE

As stated in Section 2 of the main text, the production of DMF–DrMF hybrid devices requires the deposition of a dielectric layer (Parylene C) and a hydrophobic layer (PTFE, or polytetrafluoroethylene), necessary to the DMF section of the devices. To avoid any possible disruption at the interface between moieties, which leads to electrolysis of the LAMP buffer solution (results not shown), both layers were deposited after sealing the DrMF section to the glass substrate containing the DMF electrodes. Moreover, the entrance to the DrMF microfluidic channel was not covered, also to avoid any disruption at the interface between both moieties. Thus, the deposition of the dielectric and hydrophobic layers led to a decrease in the diameter of the entrance of the microfluidic channel at the DrMF section **(**see Figure 1 b) and c) of the main text**)**. Figure S2.1 illustrates the width variation of the entrance of the microfluidic channel after deposition of the fore-mentioned layers at the flow-focusing region, according to the equations below:

$$\Delta\ channel\ width\ after\ Parylene\ C\ = \\ = Initial\ channel\ width\ -\ channel\ width\ after\ Parylene\ C\ deposition \quad (1)$$

$$\Delta\ channel\ width\ after\ PTFE\ = \\ Initial\ channel\ width\ -\ channel\ width\ after\ PTFE\ deposition \quad (2)$$

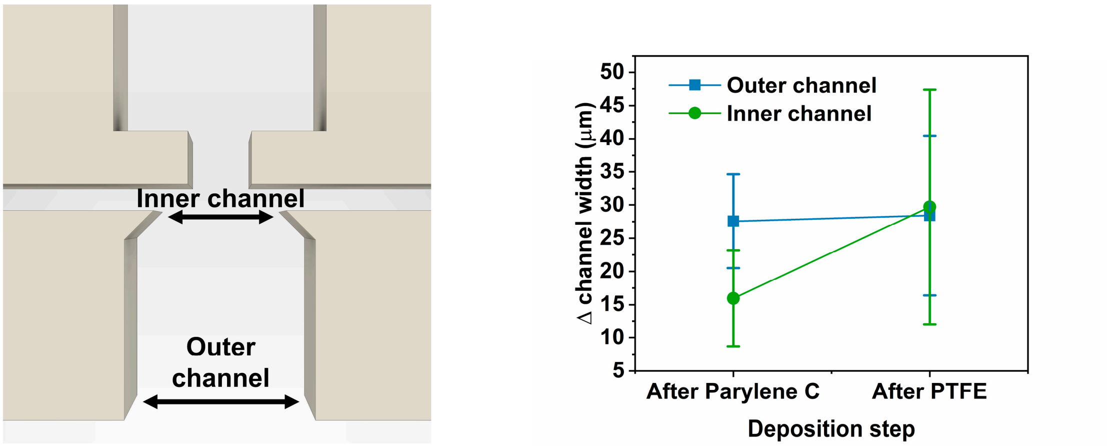

(**a**) (**b**)

Figure S2. 1: a) Schematic representation of the channel widths in DMF–DrMF devices defined for this study, i.e., the entrance of the DrMF microfluidic channel (outer channel) and the entrance to the flow-focusing region (inner channel). b) Graphical representation of the variation of the channel widths after deposition of the Parylene C layer and the PTFE layer. Error bars correspond to the standard variation.

As shown in Figure S2.1 b), both Parylene C and PTFE depositions lead to subsequent decreases in the width of the inner and outer channels of the DrMF section in hybrid devices. The inner channel is less affected by the Parylene C deposition, which leads to a larger width variation of the outer channel. This is expected, considering the conformal nature of the Parylene C deposition. In contrast, the PTFE layer is deposited by spin-coating directionally perpendicular to the microfluidic channel, which could promote a greater penetration of the PTFE solution down to the inner channel. Nevertheless, regarding the Parylene C deposition, both inner and outer channel width variations are greater than expected since the deposition process is highly conformable [1] and the deposition parameters were optimized to ensure a 2 μm thickness. Figure S2.2 illustrates one of the DMF–DrMF devices used to study the variation of channel widths, from the initial clean state to the final state, after Parylene C and PTFE depositions. The depositions were

performed exactly as described in Section 2 of the main text, and in the same order: Parylene C was first deposited with the Labcoater® PDS 2010 system, after which the PTFE layer was deposited by spin-coating. All images were acquired using a Leica M80 stereo microscope (Leica Microsystems, Wetzlar, Germany). Please note that all images are mirrored on their left side as a result of reflection from the metallic interfacial electrode. The mirrored portion of images was not cropped since the authors considered this an interesting aspect of the produced devices.

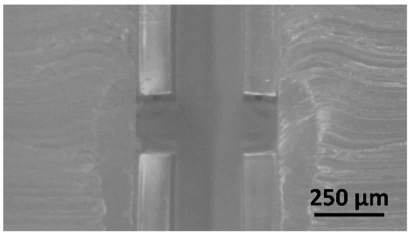

a) Outer channel before depositions

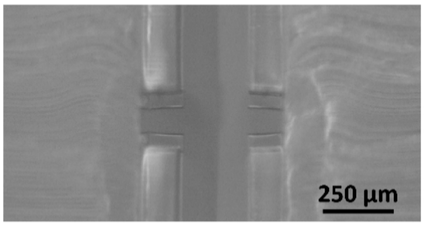

b) Inner channel before depositions

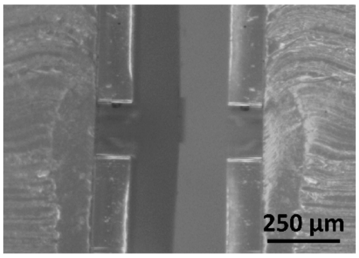

c) Outer channel after Parylene C deposition

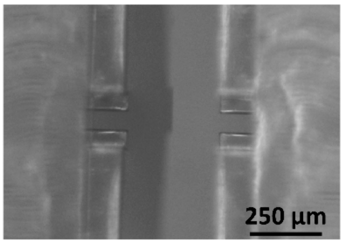

d) Inner channel after Parylene C deposition

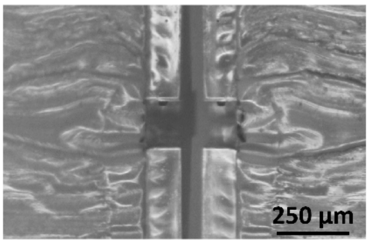

e) Outer channel after PTFE deposition

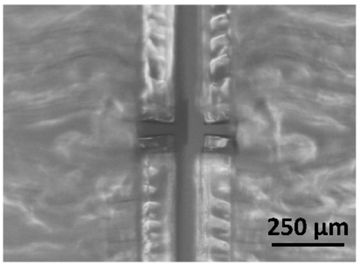

f) Inner channel after PTFE deposition

Figure S2. 2: Cross-section images of a DMF–DrMF hybrid device, evidencing the outer (left) and inner (right) channels before any deposition (a) and b), respectively), after the Parylene C deposition (c) and d), respectively), and after PTFE deposition (e) and f), respectively).

As can be seen on Figure S2.2, the deposition of Parylene C leads to a considerable reduction in the width of both outer (a) to c)) and inner (b) to d)) DrMF microfluidic channels. However, visually it seems that there was channel shrinkage rather than Parylene C deposition on the channel walls. Considering that the Parylene C is deposited in vacuum

and the PDMS is porous [2,3], the PDMS channel could have suffered shrinkage, thus decreasing both the inner and outer channels. Moreover, the silane adhesion promoter (see Section 2.2. of the main text) used in the Parylene C deposition could have induced a greater adhesion of the PDMS to the glass, further decreasing the width of microfluidic channels. After Parylene C deposition, the PTFE hydrophobic layer was deposited by spin-coating. By spin-coating, the PTFE solution is spread through the glass substrate containing both DMF and DrMF moieties (see Figure 1 a) of the main text), perpendicularly to the entrance of the DrMF microfluidic channel. An accumulation of the solution is therefore likely to occur in this area, and even though the curing process will then eliminate a considerable part of the solution solvent, the overall material accumulation should be greater in this region than for the remainder of the DMF section. Thus, the deposition of the dielectric and hydrophobic layers on the hybrid devices will lead to a decrease in the inner and outer channel widths, which will contribute to variations in droplet characteristics of the DMF–DrMF hybrid devices as opposed to standalone DrMF devices.

Supplementary Information S3

Contact angle for materials present at DMF–DrMF hybrid devices

Table S1.1 indicates the contact angles for all materials present at the interface between the DMF and the DrMF moieties of the hybrid devices, as measured in-house. Measurements were performed using an OCA15plus contact angle measurement equipment from Dataphysics (Filderstadt, Germany). Each measurement represents the average contact angle between the left and right contact angles measured for a minimum three substrates.

Table S1. 1: Contact angle measurements for multiple materials used in standalone DrMF and DMF–DrMF hybrid devices.

Material	Present in:	Contact angle (º)
PDMS (polydimethylsiloxane, Sylgard 184® from Dow Corning)	Standalone DrMF devices and DMF–DrMF devices	109.2 ∓ 1.3
Parylene C	DMF–DrMF devices	100.2 ∓ 1.0
PTFE solution used in this study	DMF–DrMF devices	122.6 ∓ 0.5

References

1. Specialty Coating Systems, SCS Parylene Properties, 2018. https://scscoatings.com/technical-library/.
2. A. Lamberti, S.L. Marasso, M. Cocuzza, PDMS membranes with tunable gas permeability for microfluidic applications. RSC Adv. **2014**, *4*, 61415–61419. https://doi.org/10.1039/C4RA12934B.
3. G. Firpo, E. Angeli, L. Repetto, U. Valbusa, Permeability thickness dependence of polydimethylsiloxane (PDMS) membranes. J Memb Sci. **2015**, *481*, 1–8. https://doi.org/10.1016/j.memsci.2014.12.043.
